# Pain Sensitization and Neuropathic Pain-like Symptoms Associated with Effectiveness of Exercise Therapy in Patients with Hip and Knee Osteoarthritis

**DOI:** 10.1155/2022/4323045

**Published:** 2022-08-29

**Authors:** Takafumi Hattori, Kazuhiro Shimo, Yuto Niwa, Yuichi Katsura, Yuji Tokiwa, Satoshi Ohga, Takako Matsubara

**Affiliations:** ^1^Faculty of Rehabilitation, Kobe Gakuin University Graduate School, Kobe, Hyogo, Japan; ^2^Department of Rehabilitation, Maehara Orthopedics Rehabilitation Clinic, Obu, Aichi, Japan; ^3^Department of Physical Therapy, Faculty of Rehabilitation, Kobe Gakuin University, Kobe, Hyogo, Japan

## Abstract

Pain sensitization and neuropathic pain-like symptoms are some of the common pain symptoms in patients with lower limbs, including hip and knee, osteoarthritis (HOA/KOA). Exercise therapy has been the first-line treatment; however, the effects differ for each patient. This prospective cohort study investigated the relationship between the effectiveness of exercise therapy and pretreatment characteristics (radiologic severity, pain sensitization, and neuropathic pain-like symptoms) of patients with HOA/KOA. We assessed the pain intensity using a numerical rating scale (NRS) before and after 12 weeks of exercise therapy in patients with HOA/KOA (*n* = 101). Before treatment, the Kellgren–Lawrence (K-L) grade; minimum joint space width (mJSW); pressure pain threshold (PPT) and temporal summation of pain (TSP) at the affected joint, tibia, and forearm; Central Sensitization Inventory-9; and painDETECT questionnaire (PDQ) were assessed. Cluster analysis was based on the pretreatment NRS and change in NRS with exercise therapy to identify the subgroups of pain reduction. The pretreatment characteristics of each cluster were compared. According to the results of the cluster analyses, patients in cluster 1 had severe pain that did not improve after exercise therapy, patients in cluster 2 had severe pain that improved, and those in cluster 3 had mild pain that improved. The patients in cluster 1 exhibited lower PPT at all measurement sites, higher TSP at the affected joint, and higher PDQ scores than those in other clusters. There was no difference in the K-L grade and mJSW among the clusters. The subgroup with severe pain and pain sensitization or neuropathic pain-like symptoms at pretreatment, even with mild joint deformity, may have difficulty in achieving improvement in pain after 12 weeks of exercise therapy. These findings could be useful for prognosis prediction and for planning exercise therapy and combining with other treatment.

## 1. Introduction

Lower limbs, including hip and knee, osteoarthritis (HOA/KOA) is the most common form of arthritis and a major cause of chronic musculoskeletal pain and disability worldwide [1, 2]. Osteoarthritis (OA) is a chronic pain disorder that is associated with nociceptive pain and structural joint damage [3]. However, pain does not necessarily correspond to osteoarthritis grade [4–6], and it is difficult to identify the joint tissue that causes the pain. For example, there are many patients with low Kellgren–Lawrence (K-L) grade who are in severe pain, and many factors contribute to their pain.

OA pain was found to be provoked by both nociceptive mechanisms and pain sensitization in the pain pathways of the central nervous system [7]. Nociceptive inputs from the joint cause peripheral sensitization, which, on occurring repeatedly, causes central sensitization [7, 8]. Quantitative sensory testing (QST), which is psychophysical testing of somatosensory function, pressure pain threshold (PPT), and temporal summation of pain (TSP) can be used to assess the pain sensitization in patients with OA [9]. Additionally, symptoms, such as fatigue, cognitive impairment, and sleep deprivation, may indicate the presence of central sensitization-related symptoms [10]. Moreover, neuropathic pain-like symptoms may occur in association with pain sensitization in OA [11–14]. Pain sensitization or neuropathic pain-like symptoms are associated with pain severity in patients with OA [9, 15, 16].

The goal of OA management is to control and improve pain, physical function, and health-related quality of life [17]. Nonpharmacological treatment is recommended for HOA/KOA [18, 19]. Specifically, exercise therapy reportedly improves pain, physical function, and the quality of life, with few adverse events [20]. However, the effect size was only moderate, as some patients with OA might not benefit from exercise therapy [20]. Therefore, it is necessary to focus on predicting exercise therapy outcomes to optimize the treatment algorithms.

Prior studies in OA have shown that pain reduction was divided into several types (based on a slow or rapid reduction in pain) and that pretreatment physical and psychosocial health factors predict the response to exercise therapy [21]. However, the potential effects of pain sensitization and neuropathic factors on pain reduction have not been investigated previously. Predicting the effectiveness of exercise therapy based on the pain mechanism is beneficial for prognosis prediction, planning exercise therapy, and combining with other treatments, such as pharmacotherapy.

The present study aimed to identify the subgroups of patients with HOA/KOA who showed similar types of pain reduction with exercise therapy and to explore the associations between the types of pain reduction and the pretreatment characteristics of pain sensitization and neuropathic pain-like symptoms. We hypothesized that pain sensitization and neuropathic pain-like symptoms would influence the effectiveness of exercise therapy.

## 2. Patients and Methods

### 2.1. Study Design

This study used a prospective cohort design. Patients with HOA/KOA, who were newly referred for physiotherapy by an orthopedic surgeon, were identified at the Maehara Orthopedic Rehabilitation Clinic in Obu, Japan, between December 1, 2019, and March 31, 2021. The inclusion criteria were as follows: a diagnosis of HOA/KOA confirmed by radiographic findings (K-L grade ≥ 1), clinical pain as the primary musculoskeletal complaint, 40 years of age or older, and chronic joint pain for at least 6 months. The exclusion criteria were rheumatoid arthritis, lumbar spinal diseases with neurological deficits and radicular pain, presence of other pain types (e.g., neck and back pain), previous hip and knee replacement, any type of surgery within the past 6 months, cognitive impairment, and severe medical comorbidities (e.g., congestive heart failure, cerebrovascular disease, and cancer). In case of bilateral symptomatic OA, the more symptomatic hip or knee was defined as the affected joint. Patients were asked to refrain from normal exercise on the day they participated in the study procedure.

### 2.2. Protocol

Demographic data, radiologic findings, pain intensity within the last 24 hours (numerical rating scale (NRS), 0 to 10) [22], PPT and TSP, Central Sensitization Inventory-9 (CSI-9) data, and painDETECT Questionnaire (PDQ) data were recorded before treatment. Pain intensity was reassessed after 12 weeks of exercise therapy.

### 2.3. Demographic Data

The patients were interviewed to assess demographic characteristics (age, sex, body mass index [BMI], pain duration, pain symptoms, and pain-related disability). Pain symptoms and pain-related disability were assessed using subscales of pain and function in activity of daily living in the Hip Disability and Osteoarthritis Outcome Score (HOOS) [23, 24] or the Knee Injury and Osteoarthritis Outcome Score (KOOS) [25, 26]. HOOS and KOOS were found to be reliable and valid for patients with HOA/KOA, with scores ranging from 0 (worst) to 100 (best) for each subscale.

### 2.4. Radiologic Severity

Posteroanterior radiographs of the hip and knee with weigh bearing were assessed. The K-L grade [27] and minimum joint space width (mJSW) [28] were assessed by a single orthopedic surgeon. The characteristics for each K-L grade can be summarized as follows: grade 1, doubtful OA, with the presence of minor osteophytes of doubtful importance; grade 2, minimal OA, with definite osteophytes but an unimpaired joint space; grade 3, moderate OA, with osteophytes and a moderate diminution of the joint space; and grade 4, severe OA, with a greatly impaired joint space and sclerosis of the subchondral bone.

### 2.5. Mechanistic Pain Profiling

QST was assessed at the affected joint, the tibialis anterior (tibia, 5 cm distal to the tibial tuberosity), and the extensor carpi radialis longus (forearm, 5 cm distal to the lateral epicondyle of the humerus) [15, 29]. The four sites on the hip and knee were located as follows: 3 cm proximal to the tip of the greater trochanter (Hip-1), 3 cm posterior to the posterior edge of the greater trochanter (Hip-2), 3 cm distal to the distal edge of the greater trochanter (Hip-3), 3 cm anterior to the anterior edge of the greater trochanter (Hip-4), 2 cm distal to the inferior medial edge of the patella (Knee-1), 2 cm distal to the inferior lateral edge of the patella (Knee-2), 3 cm lateral to the mid-point on the lateral edge of the patella (Knee-3), and 3 cm medial to the mid-point on the medial edge of the patella (Knee-4) [15, 29].

PPT and TSP were measured by a physiotherapist using a handheld pressure algometer (Algometer Type II, Somedic AB, Sweden) with a 1 cm^2^ probe, in the following order at 5-minute intervals: affected joint, tibia, and forearm. The pressing rate was 30 kPa/s; two assessments were performed at each site, and the mean PPT value was used. The PPT of the affected joint was defined as the lowest PPT of the four sites in the affected joints [15, 16].

TSP is a method for assessing central sensitization by repeated pain stimulus [15]. Pressure stimulations comprised 10 stimuli at PPT level, with 1-s duration and 1-s interval [15, 16]. Patients rated the pain intensity during consecutive stimulations on a visual analog scale (VAS), where “0” indicated “no pain,” and “100” indicated the “worst possible pain.” Skin contact between stimulations was maintained at a painless level to prevent displacement of the stimulation site. TSP was calculated by subtracting the pain rating at the first stimulus from that at the tenth stimulus [30]. TSP at the affected joint was assessed at the most sensitive of the four PPT sites.

### 2.6. Central Sensitization-Related Symptoms

CSI-9 was used to assess the central sensitization-related symptoms [31]. The CSI-9, with nine items, is a short version of the 25-item CSI. A total score of 0–9 is classified as “subclinical,” 10–19 is classified as “mild,” and 20–36 is classified as “moderate/severe.”

### 2.7. Neuropathic Pain-like Symptoms

The PDQ was used to identify factors related to neuropathic pain [32]. The PDQ is a self-report questionnaire with nine items [32] and comprises seven sensory descriptor items and two items related to the spatial and temporal characteristics. A total PDQ score of -1 to 12 is classified as “nociceptive,” 13–18 as “unclear,” and 19–38 as “neuropathic.”

### 2.8. Intervention

The participants received standard exercise therapy that was individualized and supervised by physiotherapists at the participating clinic. Physiotherapists who participated in the study had joined a workshop on the pathogenesis of pain in OA and the recommended nonpharmacological treatments based on clinical practice guidelines. The recommended treatment included exercise programs that were individualized and progressive, considering the preferences and ability of the patients [18]. The exercise therapy lasted approximately 40 minutes per session and comprised the following components: strength exercises; active range of motion exercises; aerobic exercises, including walking and cycling; and neuromuscular exercises for the trunk, hips, and knees [18, 19, 33]. The exercises in the clinic were carried out once a week for 12 weeks, and the patients were instructed to perform the exercises at home for at least 3 days a week. Recent studies recommend 8–12 weeks of continuous exercise therapy in patients with OA [34–38]. The physiotherapist verified the implementation of patients' home exercises from their self-record sheets during the clinic visits. During the intervention period, patients were monitored for adverse events and analgesic use. The physiotherapist instructed them on exercises and lifestyle modifications to reduce hip/knee joint loading when their pain increased. In addition, patient education and advice on self-management strategies were provided in accordance with the clinical guidelines [19].

### 2.9. Statistical Analysis

Data were presented as mean ± standard deviation (SD). Patient subgroups were formed using hierarchical agglomerative cluster analysis. Hierarchical agglomerative cluster analysis (Ward's method) was performed using the pretreatment NRS and the amount of change in the NRS from pretreatment to after 12 weeks to identify the pain reduction subgroup according to the distribution of pretreatment pain intensity and amount of change. The number of clusters was determined using gap values [39]. Following the formation of clusters, the Kruskal–Wallis test and Fisher's exact test were used to compare variables; the post hoc Steel–Dwass test was used to compare the differences between each cluster. For comparisons between the pretreatment and posttreatment NRS, Wilcoxon signed-rank test was applied. We also used “*R*” as calculated by *Z* translation to evaluate the magnitude of the effect size (*r* = *Z*/√*N*) [40]. As an additional analysis, we analyzed the differences between HOA and KOA within clusters for each assessment. Statistical analyses were performed using SPSS (version 27.0, IBM Corporation, Armonk, NY, USA) and *R* (version 3.3.0). The significance level was set at *P* < 0.05.

## 3. Results

**Figure 1:**
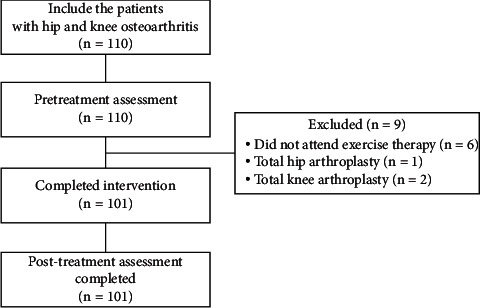
Participant flow through study.

Of the 110 patients (HOA, *n* = 46; KOA, *n* = 64) who received a diagnosis of HOA/KOA, 9 patients (HOA, *n* = 4; KOA, *n* = 5) were excluded because they did not attend the follow-up visit owing to personal reasons (*n* = 6), total hip replacement (*n* = 1), or total knee replacement (*n* = 2). There were 101 patients in this study, and the participant flowchart is shown in Figure 1. The patients who were excluded were not significantly different from those who were included in terms of age (*P* = 0.608), sex (*P* = 0.680), BMI (*P* = 0.257), pain intensity (*P* = 0.624), or pain duration (*P* = 0.204). There were no adverse events due to this exercise therapy.  Table 1 shows analgesic drugs used continuously during the intervention period. When their pain decreased during the exercise period, analgesic prescriptions were reduced or discontinued. In the additional analysis, all demographics in each cluster were not significantly different between HOA and KOA.

### 3.1. Types of Pain Reduction according to the Cluster Analysis

**Figure 2:**
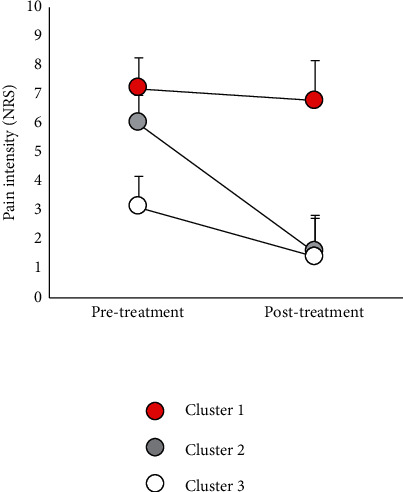
Types of pain reduction of each cluster. Data are presented as mean ± SD (*n* = 101). NRS, numerical rating scale.

Cluster analysis using the pretreatment NRS and amount of change in the NRS between pretreatment and after 12 weeks yielded three clusters. The demographic data are shown in Table 1 and the types of pain reduction in Figure 2. Of the 101 patients (HOA: *n* = 42, KOA: *n* = 59) who were assessed at 12 weeks after pretreatment, 28 (HOA: *n* = 13, KOA: *n* = 15) were categorized into cluster 1, 19 (HOA: *n* = 4, KOA: *n* = 15) into cluster 2, and 54 (HOA: *n* = 25, KOA: *n* = 29) into cluster 3. There was no significant difference in the proportions of HOA and KOA (*P*=0.135). The pretreatment NRS in clusters 1 (7.2 ± 1.0, *R* = 0.810, *P* < 0.001) and 2 (6.0 ± 1.0, *R* = 0.523, *P* < 0.001) were higher than those in cluster 3 (3.1 ± 1.1). The pretreatment NRS in clusters 1 and 2 were not significantly different (*P*=0.280). The posttreatment NRS after exercise therapy in cluster 1 (6.8 ± 0.4) was higher than those in clusters 2 (1.6 ± 1.3, *R* = 0.544, *P* < 0.001) and 3 (1.4 ± 1.3, *R* = 0.739, *P* < 0.001). There was no significant difference in the posttreatment NRS between clusters 2 and 3 (*P*=1.000).

A significant reduction in pain was observed in clusters 2 (*R* = 0.559, *P* < 0.001, rate of pain reduction: 73.7%) and 3 (*R* = 0.524, *P* < 0.001, 55.4%) after exercise therapy, while cluster 1 did not show any significant reduction (*P* = 0.059, 5.9%). The amount of change in the NRS from pretreatment to after 12 weeks in cluster 1 (0.4 ± 1.0) was lower than the amounts in clusters 2 (4.4 ± 1.4, *R* = 0.666, *P* < 0.001) and 3 (1.7 ± 1.4, *R* = 0.326, *P* < 0.001). In addition, the amount of change in the NRS in cluster 2 was higher than that in cluster 3 (*R* = 0.458, *P* < 0.001). According to the results of the cluster analyses, patients in cluster 1 had severe pain that did not improve after exercise therapy; patients in cluster 2 had severe pain that improved, and those in cluster 3 had mild pain that improved.

### 3.2. Radiographic Assessment

The results of radiographic assessments are shown in Table 2. There were no differences among clusters 1, 2, and 3 (*P*=0.629) in the percentage of patients with each K-L grade. There were no significant differences in the mJSW among clusters 1, 2, and 3 (*P*=0.477). Additional analysis showed that radiographic findings in each cluster were not significantly different between HOA and KOA.

### 3.3. Mechanistic Pain Profiling

**Figure 3:**
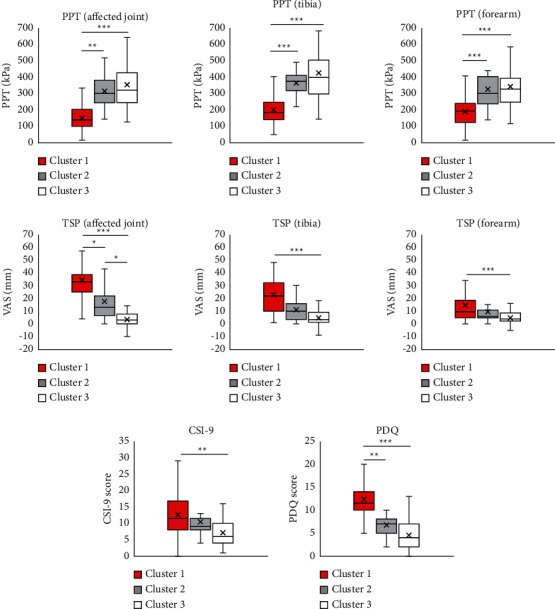
Pressure pain threshold, temporal summation of pain, Central Sensitization Inventory-9, and painDETECT Questionnaire of each cluster. Data are presented as mean ± SD (*n* = 101). The symbols ^*∗*^, ^*∗∗*^, and ^*∗∗∗*^indicate significant difference between clusters (*P* < 0.05, 0.01, 0.001, respectively). PPT, pressure pain threshold; TSP, temporal summation of pain; CSI-9, Central Sensitization Inventory-9; PDQ, painDETECT Questionnaire; VAS, visual analog scale.

The PPT values for each cluster are shown in Figure 3. The mean PPT at the affected joint in cluster 1 (149.5 ± 69.6) was lower than the values in clusters 2 (313.7 ± 117.9, *R* = 0.433, *P* < 0.001) and 3 (351.3 ± 166.8, *R* = 0.627, *P* < 0.001). The mean PPT values at the affected joint in clusters 2 and 3 were not significantly different (*P* = 1.000). The PPT at the tibia in cluster 1 (198.5 ± 91.0) was lower than the values in clusters 2 (362.4 ± 80.0, *R* = 0.403, *P* < 0.001) and 3 (422.5 ± 180.3, *R* = 0.606, *P* < 0.001). The mean PPT values at the tibia in clusters 2 and 3 were not significantly different (*P* = 1.000). At the forearm, the mean PPT in cluster 1 (189.8 ± 85.3) was lower than the values in clusters 2 (326.4 ± 126.1, *R* = 0.381, *P* < 0.001) and 3 (342.0 ± 134.0, *R* = 0.536, *P* < 0.001). The mean PPT at the forearm were not significantly different between clusters 2 and 3 (*P* = 1.000).

The TSP values for each cluster are shown in Figure 3. At the affected joint, the mean TSP in cluster 1 (34.2 ± 14.9) was higher than the values in clusters 2 (17.4 ± 14.0, *R* = 0.261, *P* < 0.05) and 3 (3.6 ± 5.5, *R* = 0.759, *P* < 0.001). The mean TSP in cluster 2 was higher than the values in cluster 3 (*R* = 0.372, *P* < 0.05). At the tibia, the mean TSP between clusters 1 (22.6 ± 15.8) and 2 (11.1 ± 9.3) were not significantly different (*P* = 0.058). Moreover, the mean TSP in cluster 3 (4.6 ± 7.5) was lower than that in cluster 1 (*R* = 0.549, *P* < 0.001) and was not significantly different from that in cluster 2 (*P* = 0.058). At the forearm, the mean TSP in clusters 1 (14.4 ± 14.8) and 2 (9.5 ± 9.9) were not significantly different (*P* = 1.000). The mean TSP in cluster 3 (5.7 ± 6.1) was lower than that in cluster 1 (*R* = 0.296, *P* < 0.01) and was not significantly different from that in cluster 2 (*P* = 0.362).

According to additional analysis, the mean PPT and TSP at the affected joint, tibia, and forearm were not significantly different between HOA and KOA in clusters 1 and 3. In cluster 2, mean PPT (HOA: affected joint: 350.0 ± 134.0, tibia: 370.0 ± 67.3, forearm: 336.8 ± 94.2; KOA: affected joint: 304.1 ± 120.6, tibia: 360.4 ± 87.8, forearm: 323.7 ± 140.2) and TSP (HOA: affected joint: 16.8 ± 7.0, tibia: 7.8 ± 8.8, forearm: 11.6 ± 13.3; KOA: affected joint: 17.5 ± 16.0, tibia: 11.9 ± 9.8, forearm: 8.6 ± 7.9) at all measurement sites were approximately the same level in both HOA and KOA. However, PPT and TSP could not be statistically analyzed due to the small sample size.

### 3.4. Central Sensitization-Related Symptoms

The CSI-9 scores for each cluster are shown in Figure 3. The mean CSI-9 score in cluster 1 (12.6 ± 7.4) was not significantly different from that in cluster 2 (10.5 ± 5.9, *P*=0.882) and was significantly higher than that in cluster 3 (7.2 ± 4.5, *R* = 0.348, *P* < 0.01). CSI-9 levels were not significantly different between clusters 2 and 3 (*P*=0.174). In cluster 1, 10 patients (36%) were classified as subclinical, 12 (43%) as mild, and 6 (21%) as moderate/severe. In cluster 2, 12 patients (63%) were classified as subclinical, 5 (26%) mild, and 2 (11%) moderate/severe. In cluster 3, 39 patients (72%) were classified as subclinical, 14 (26%) mild, and 1 (2%) moderate/severe. CSI-9 scores in each cluster were not significantly different between HOA and KOA in the additional analysis.

### 3.5. Neuropathic Pain-like Symptoms

The PDQ scores for each cluster are shown in Figure 3. The mean PDQ score in cluster 1 (12.4 ± 4.5) was higher than those in clusters 2 (6.7 ± 2.9, *R* = 0.350, *P* < 0.01) and 3 (4.5 ± 3.1, *R* = 0.681, *P* < 0.001). Moreover, the mean PDQ scores were not significantly different between clusters 2 and 3 (*P*=0.117). In cluster 1, 16 patients (57%) were classified as nociceptive, 9 (32%) as unclear, and 3 (32%) as neuropathic. In cluster 2, 18 patients (95%) were classified as nociceptive, 1 (5%) was classified as unclear, and none (0%) was classified as neuropathic. In cluster 3, 53 patients (98%) were classified as nociceptive, 1 (2%) as unclear, and none (0%) as neuropathic. PDQ scores in each cluster were not significantly different between HOA and KOA, according to additional analysis.

## 4. Discussion

In this study, pain reduction by exercise therapy in patients with HOA and KOA was divided into several types. Patients with severe pain had a longer pain duration and more severe pain symptoms, as well as more severe pain sensitization, central sensitization-related symptoms, and neuropathic pain-like symptoms than patients with mild pain. Moreover, the patients with severe pain that did not improve after exercise therapy had lower PPT values at all measurement sites, facilitated TSP at the affected joint, and higher PDQ scores. These findings indicate that pain sensitization and neuropathic pain-like symptoms are associated with the effectiveness of exercise therapy.

Exercise therapy is recommended as a first-line treatment for painful OA [18, 19]. However, according to our cluster analyses, patients in cluster 1 had severe pain that did not improve after exercise therapy, patients in cluster 2 had severe pain that improved, and patients in cluster 3 had mild pain that improved. Pain reduction of 30% or 50% has been defined as the criterion for response to treatment. In the present study, the rates of pain reduction were 5.9%, 73.7%, and 55.4% in clusters 1, 2, and 3, respectively. Thus, patients in clusters 2 and 3 were considered responders to exercise therapy, whereas those in cluster 1 were considered nonresponders. In a study on exercise intervention, Lee et al. showed that KOA with severe pain comprised two types of cases: patients with no reduction in pain symptoms and those with delayed reduction [21]. Similarly, we found different responses to exercise therapy in clusters 1 and 2, although there was no difference in the pain intensity before treatment. This suggests that it is difficult to predict prognosis based on pain intensity alone and that different mechanisms may be involved in pain reduction, even when pain intensity is similar between patient groups before treatment.

OA is a chronic pain disorder involving nociceptive pain and structural joint damage. However, cross-sectional studies have shown that pain is not necessarily related to radiologic severity in patients with HOA/KOA [4, 5]. The current study showed no differences in K-L grade and mJSW between the clusters, and joint deformity was not necessarily involved in determining the types of pain reduction. Similarly, Lee et al. reported that joint deformity was not involved in the different types of pain reduction by exercise therapy in patients with KOA [21]. In addition, the K-L grade of 2 or higher is a common inclusion criterion for many OA studies [41, 42]; however, in the present study, we included patients with the K-L grade of 1. Although our results were only based on the effectiveness of a short-term intervention, we suggest that patients with severe pain may not show pain reduction after 12 weeks of exercise therapy, despite mild joint deformity.

Several studies have reported that pain sensitization is associated with OA pain [15, 16]. Lower PPT and facilitated TSP were observed in the affected joint and in remote sites in patients with painful HOA and KOA [15, 16]. Other studies have shown the impact of pain sensitization on postsurgical pain in patients with OA [29, 30, 43, 44]. Regarding the predictive role of exercise therapy, the lower PPT and facilitated TSP emerged as robust predictors of nonresponse to physiotherapy for 6 months in patients with KOA [45]. In our study, PPT and TSP were measured systemically; PPT was lower in the affected joint, tibia, and forearm, and TSP in the affected joint was higher in cluster 1 than in cluster 2. The PPT at the affected site indicates peripheral sensitization, while lower PPT at the remote site and facilitated TSP reflect central sensitization. Thus, our results indicate that both peripheral and central sensitization may predict response to exercise therapy.

Additionally, our study found differences in neuropathic pain-like symptoms at pretreatment, according to the type of pain reduction between each cluster. Recent studies have reported that neuropathic pain-like symptoms were quite prevalent in patients with HOA and KOA; respectively, 37% and 46% experienced at least one neuropathic phenotype [11–14]. These results suggest that pain sensitization and neuropathic pain-like symptoms, despite minor changes on radiographic imaging, may be associated with nonresponse to exercise therapy in patients with HOA and KOA.

Our study showed that while the CSI-9 in cluster 1 was higher than that in cluster 3, it did not differ from that in cluster 2. Central sensitization-related symptoms are a characteristic of chronic pain disorders, such as fibromyalgia, chronic widespread pain, and low back pain [46]. However, a recent study by Mibu et al. reported that CSI was not involved in the pain in KOA [47]. Additionally, a previous study by OʼLeary et al. suggested that the PPT and TSP could predict prognosis better than CSI in KOA [45]. Our results suggest that there may be a difference in central sensitization-related symptoms between OA with severe pain and OA with mild pain; however, it may be difficult to predict the prognosis of pain using the CSI-9 in patients with severe pain at pretreatment.

This study has several limitations. First, the participants in this study were HOA/KOA patients for whom physiotherapy was prescribed by orthopedic surgeons; this may have led to selection bias. Second, although our results showed no difference in the distribution in each subgroup, HOA and KOA may exhibit different characteristics. However, in this study, there were no significant differences in characteristics between HOA and KOA patients among the clusters. Furthermore, a recent cohort study (*n* = 32,599) has shown similar effects of exercise therapy on pain reduction in HOA and KOA patients [37]. Therefore, this study may provide new insights into the influence of pain sensitization and neuropathic pain-like symptoms on the effectiveness of exercise therapy in both HOA and KOA. Third, the K-L grade and mJSW were used to evaluate radiographic changes, but not joint inflammation, meniscus tear, and hip labral tear. For example, lower PPT at the affected joint in KOA is related to the degree of synovitis [48]. Thus, it is possible that joint inflammation and other pathologies were involved in delayed pain reduction in the patients in our study. Future studies should confirm the characteristics of structural joint changes using magnetic resonance imaging. Finally, we did not assess physical activity in daily living. Although there is no conclusive evidence indicating that high physical activity increases the pain-inhibitory effect of exercise [49], it is possible that the amount of physical activity affects pain reduction. Additional studies are needed to examine the effect of exercise therapy with monitoring of physical activity.

## 5. Conclusions

Our findings suggest that pain did not improve in HOA and KOA patients with severe pain symptoms, pain sensitization, and neuropathic pain-like symptoms after exercise therapy for 12 weeks despite mild joint deformity. However, exercise therapy may be effective in improving pain in patients who have severe pain without prominent pain sensitization and neuropathic pain-like symptoms. Thus, assessment of pain sensitization and neuropathic pain-like symptoms is required for adequate treatment selection and prognosis prediction. Future studies are needed to examine the effectiveness of pharmacotherapy and exercise interventions that primarily address pain sensitization and neuropathic factors in patients with OA who exhibit a low response to treatment.

## Figures and Tables

**Table 1:** Demographic data of each cluster.

Variables	Cluster 1 (*N* = 28, 28%)	Cluster 2 (*N* = 19, 19%)	Cluster 3 (*N* = 54, 53%)	*P*
Type of OA				
HOA, *n* (%)	13 (46)	4 (21)	25 (47)	0.135
KOA, *n* (%)	15 (54)	15 (79)	29 (53)	

Age, mean ± SD	63.8 ± 14.1	64.6 ± 10.5	62.7 ± 11.6	0.845
Females, *n* (%)	25 (89)	17 (89)	40 (74)	0.327
BMI, mean ± SD (kg/m^2^)	24.2 ± 2.6	25.5 ± 4.2	24.0 ± 3.0	0.488
Pain duration, mean ± SD (month)	71.7 ± 53.5^ab^	37.6 ± 38.5	40.4 ± 49.1	<0.01

HOOS, KOOS				
Pain symptoms, mean ± SD (0–100)	47.1 ± 16.7^ab^	63.5 ± 13.0^c^	74.7 ± 14.8	<0.001
Function of ADL, mean ± SD (0–100)	55.0 ± 18.8^ab^	75.2 ± 11.5^c^	84.8 ± 12.6	<0.001

Analgesic use				
NSAIDs, *n* (%)	22 (79)	8 (42)	19 (35)	<0.001
Duloxetine, *n* (%)	5 (18)	0 (0)	0 (0)	<0.001
Tramadol, *n* (%)	3 (11)	0 (0)	0 (0)	<0.05

There were significant differences in pain duration, pain symptoms, disability, and analgesic use between each cluster. ^a^Significant group difference between clusters 1 and 2 (*P* < 0.05, Bonferroni). ^b^Significant group difference between clusters 1 and 3 (*P* < 0.05, Bonferroni). ^c^Significant group difference between clusters 2 and 3 (*P* < 0.05, Bonferroni). OA, osteoarthritis; HOA, hip osteoarthritis; KOA, knee osteoarthritis; BMI, body mass index; NRS, numerical rating scale; HOOS, Hip Disability and Osteoarthritis Outcome Score; KOOS, Knee Injury and Osteoarthritis Outcome Score; ADL, activities of daily living; NSAIDs, nonsteroidal anti-inflammatory drugs.

**Table 2:** Radiographic assessment of each cluster.

Variables	Cluster 1	Cluster 2	Cluster 3	*P*
K-L grade				
1, *n* (%)	9 (32)	9 (47)	21 (39)	0.629
2, *n* (%)	6 (22)	3 (16)	15 (28)	
3, *n* (%)	9 (32)	5 (26)	13 (24)	
4, *n* (%)	4 (14)	2 (11)	5 (9)	

mJSW, mean ± SD	3.3 ± 1.2	2.9 ± 1.4	2.9 ± 1.6	0.477

There were no significant differences in K-L grade and mJSW between each cluster. HOA, hip osteoarthritis; KOA, knee osteoarthritis; K-L, Kellgren–Lawrence; mJSW, minimum joint space width.

## Data Availability

The data underlying the findings described in this manuscript are available from the corresponding author upon reasonable request.
